# Osimertinib‐induced severe interstitial lung disease: A case report

**DOI:** 10.1111/1759-7714.13127

**Published:** 2019-06-26

**Authors:** Mengdi Fan, Ting Mo, Lulei Shen, Li Yang

**Affiliations:** ^1^ Department of Endocrinology Shu Lan (HangZhou) Hospital Hangzhou China; ^2^ Department of Respiratory Shu Lan (HangZhou) Hospital Hangzhou China

**Keywords:** Carcinoma of the lung, osimertinib, interstitial lung disease, prognosis

## Abstract

Lung cancer is globally one of the leading causes of malignant tumor‐related mortality and ranks as having the highest incidence found in men and second in women. Osimertinib is a drug used to target lung cancer but its toxicity is not fully understood. Here we present a case of lung adenocarcinoma in a 78‐year‐old man that was treated and responded favorably with Osimertinib after the failure of other therapies. Unfortunately, the patient developed severe interstitial lung disease during the treatment procedure.

## Introduction

Lung cancer is the leading cause of cancer associated death and ranks as having the highest incidence found in men and second in women.^1^ The morbidity and mortality of lung cancer has increased in recent years. Statistics indicate that from 2006 to 2011, the incidence of lung cancer was 13/100000 in the general population.^2^ Non‐small cell lung cancer (NSCLC) is the main form of lung cancer accounting for 80–85% of all lung carcinomas. Although, the treatment modality of NSCLC has developed rapidly with the introduction of drugs designed to target this disease, the prognosis of NSCLC is still to be determined.^3^

The target drug, epidermal growth factor receptor (EGFR) tyrosine kinase inhibitors (TKIs) are widely used in the treatment of NSCLC and satisfactory results have been previously reported.^4^ The overall survival (OS) and disease free survival (DFS) was found to be significantly increased in patients who received EGFR‐TKIs compared to chemotherapies for EGFR mutated patients. However, within six months to one year, drug resistance to EGFR‐TKIs multi‐therapy appears to be unavoidable. How to manage those patients who are resistant to EGFR‐TKI therapy remains a challenge for clinicians.

In November 2015, the United States Food and Drug Administration (FDA) approved the clinical use of osimertinib treatment for NSCLC patients with EGFR T790M positive mutation and drug resistance after the first and second generation of EGFR‐TKI treatment^5^ However, drug‐related toxicity is not clearly understood when osimertinib is only administered for a short period. In our study, we present a case treated with osimertinib where a complete response was achieved after other therapies had failed. During the treatment procedure, the patient developed severe interstitial lung disease.

## Case report

**Figure 1:**
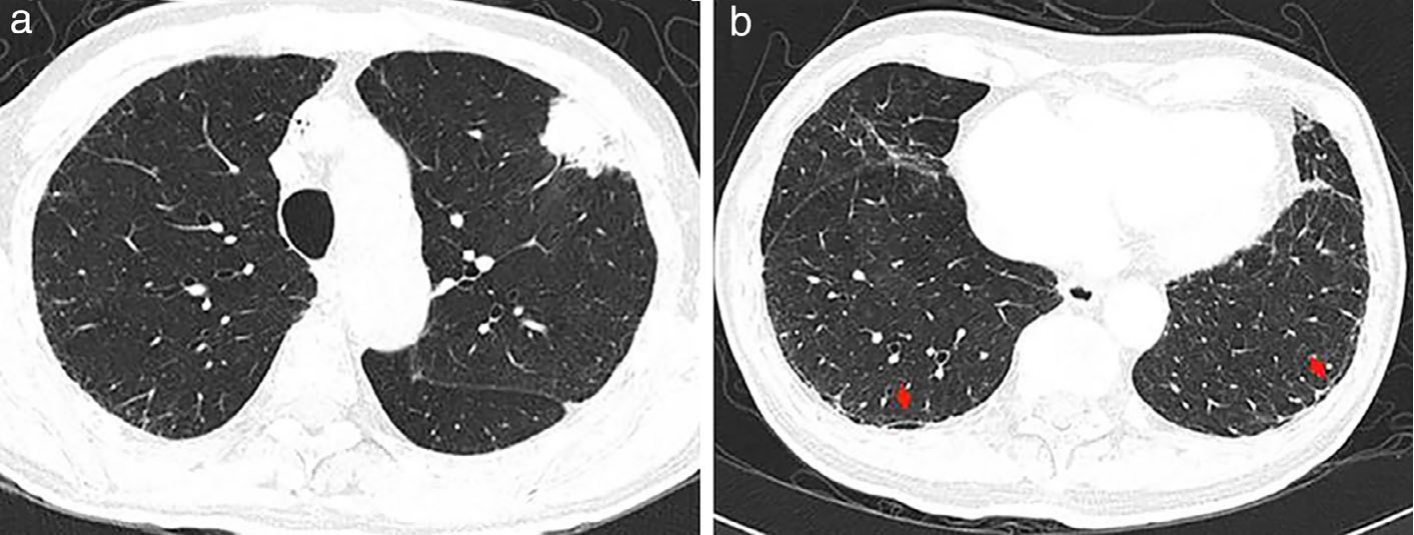
CT scan of the chest showed a pulmonary space‐occupying lesion in the left upper lobe highly suspicious of lung cancer. (**a**) A pulmonary space‐occupying lesion is visible in the left upper lobe. (**b**) Bilateral subpleural line of the lung indicated by red arrows.

**Figure 2:**
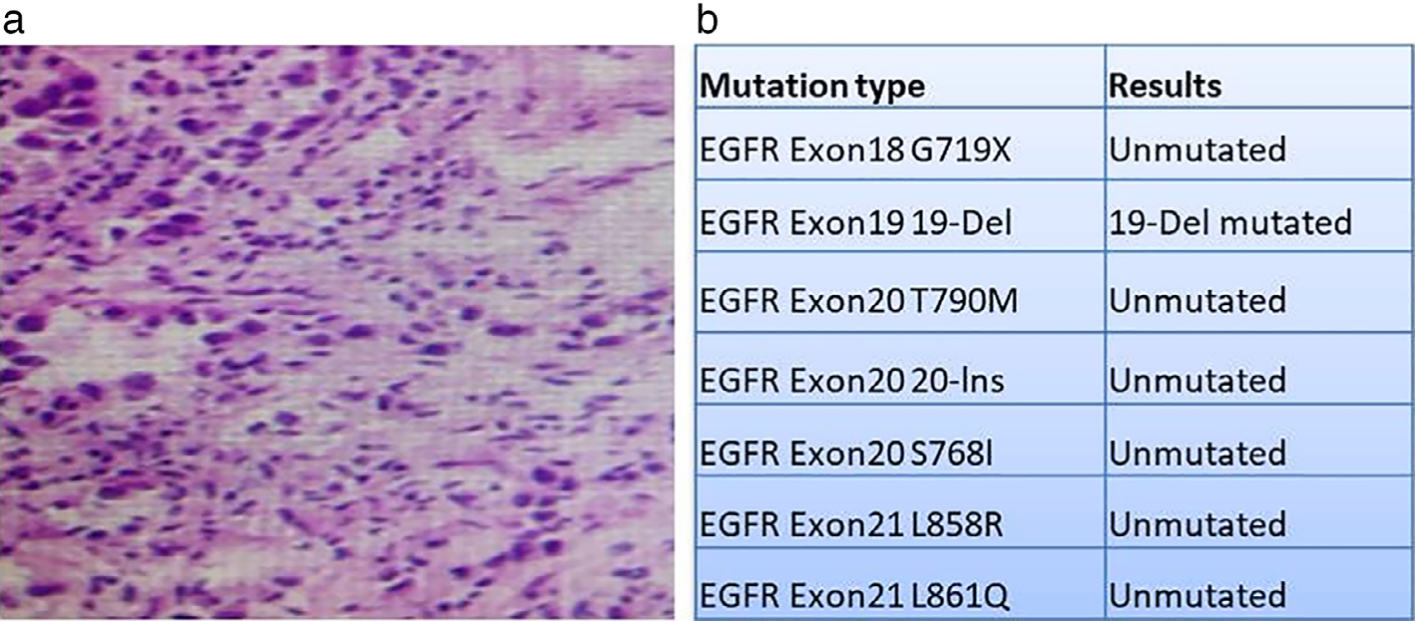
Following fine needle biopsy, pathology confirmed a diagnosis of lung adenocarcinoma with EGFR mutation. (**a**) Pathology demonstrated lung adenocarcinoma, original magnification ×200. (**b**) Indicates EGFR exon 19 mutation with 19‐deletion (19‐Del) .

**Figure 3:**
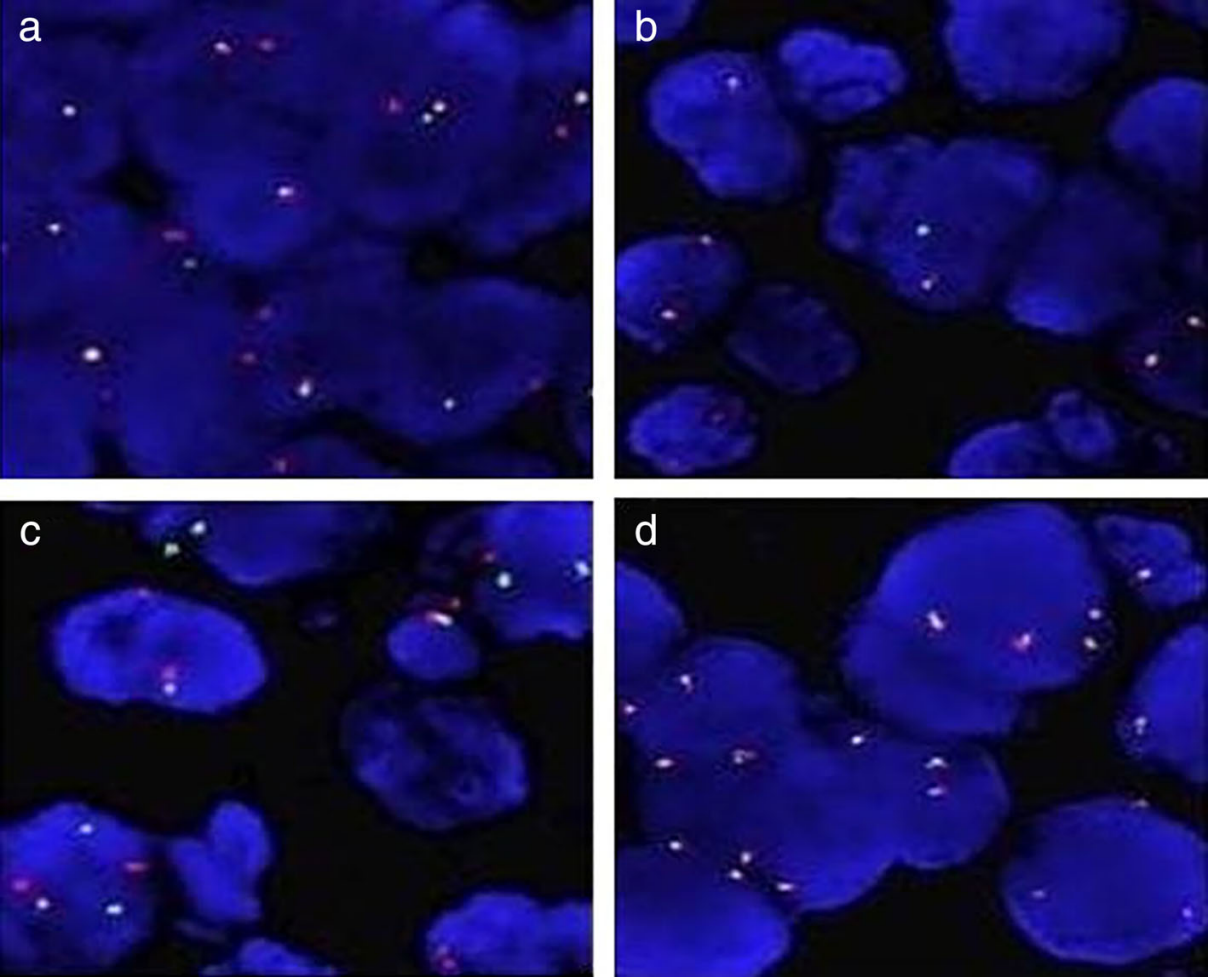
Immunofluorescence assay indicated the ALK ROS1 was negative. (**a**) ALK control. (**b**) Case of the ALK examination. (**c**) Control of ROS1 examination. (**d**) Case of the ROS1 examination.

**Figure 4:**
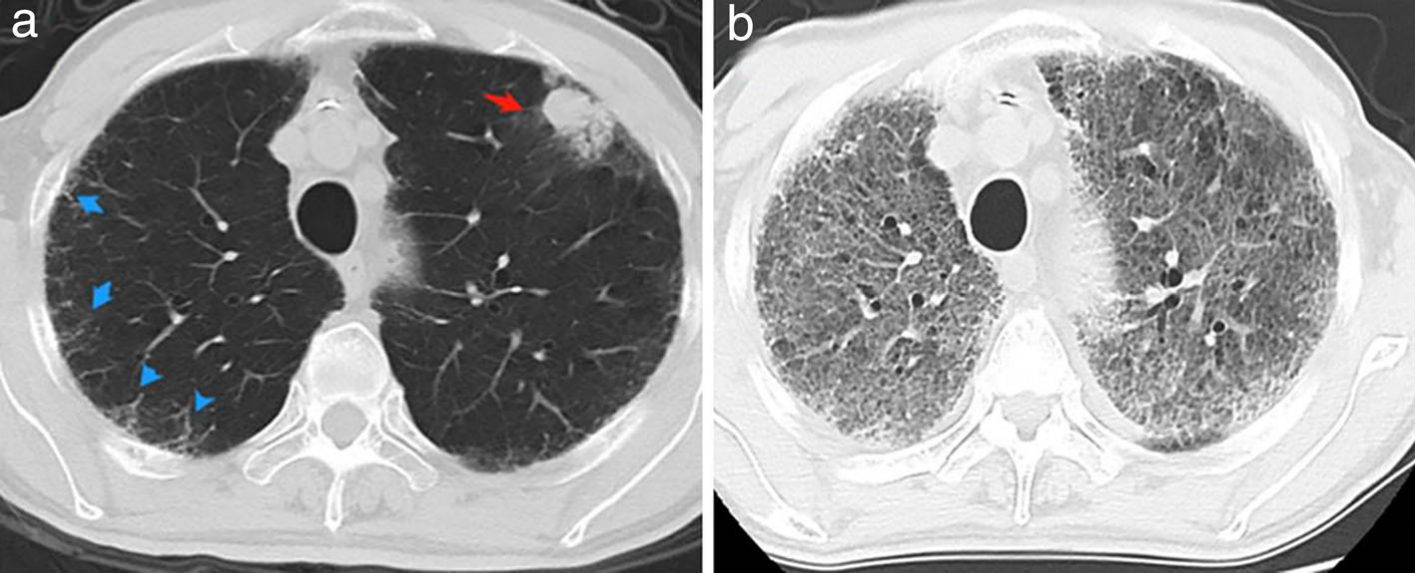
Chest CT before and after osimertinib treatment. (**a**) A round space‐occupying lesion with surrounding ground‐glass opacity was seen (indicated by red arrow) before osimertinib treatment. (**b**) A complete response after osimertinib treatment was achieved although severe interstitial lung disease was visible.

A 78‐year‐old male patient presented in April 2015 with suspected lung cancer. Chest CT examination revealed a pulmonary space‐occupying lesion in the left upper lobe of the lung (Fig. 1). Following fine needle biopsy, pathology confirmed the patient had lung adenocarcinoma and EGFR (Fig. 2), ALK and ROS1 were negative (Fig. 3). Due to the advanced age of the patient (78) together with a diagnosis of atrial fibrillation, EGFR‐TKIs was recommended as the treatment of choice. From 23 October 2015, the patient commenced gefitinib 250 mg orally once a day until the disease progressed in July 2016 when CT scan of the chest indicated metastasis in the lung. The gefitinib, everolimus combined therapy was subsequently recommended because of disease progression. However, after a few months of combined treatment, everolimus had to be withdrawn because the patient experienced severe breathlessness, cough and mouth ulcers. At that time, the patient had been taking gefitinib for 16 months until drug resistance developed on 16 February 2017 (Fig. 4(a)).

The patient had second gene mutation testing at this time which indicated EGFR T790M mutation. Osimertinib 80 mg/day orally was subsequently recommended from March 2017. After only one month of treatment, chest CT scan revealed the disease had almost completely resolved (Fig. 4(b)). However, severe interstitial lung disease was also confirmed (Fig. 4(b)). The patient was found to have severe cough and difficulty in breathing at this time and the symptoms did not resolve, even after anti‐infection and anti‐cough treatments were administered. The patient attended our hospital for further treatment. After reviewing his treatment history and chest xray, we considered that he was suffering from severe drug‐induced interstitial lung disease and advised him to cease taking the osimertinib immediately. Unfortunately, he still continued to take the osimertinib as he considered the medicine to be effective for his tumor, and refused to tell the doctor the truth until it was found that he was suffering from further dyspnea. After anti‐infection and high dosage methylprednisolone (240 mg/day) and mechanical ventilation, the patient finally died of multiple organ failure after 2 weeks treatment for severe interstitial lung disease and resultant complications.

## Discussion

Lung cancer is the main cause of cancer deaths. Smoking, air pollution exposure, tobacco consumption and other factors can all contribute to lung carcinogenesis. However, the general prognosis of lung cancer is poor because the disease is usually well advanced when it is first diagnosed. In the advanced stages of lung cancer, chemoradiation and target drugs are recommended. If the decision is made to use first generation target drugs (gefitinib and Erlotinib), EGFR mutation status examination is necessary. Overall survival and disease free survival can be significantly prolonged for patients with effective EGFR mutation who are treated with gefitinib or Erlotinib. However, drug resistance developed in most cases that received 9–13 months treatment. In the case reported here, the patient developed gefitinib resistance after 10 months administration. Previous studies have determined that multiple mechanisms of acquired resistance include secondary mutations in EGFR, ERBB2 amplification, MET oncogene amplification, etc.^6^, ^7^ The most common mechanism of target drug resistance is the EFGR T790M mutation in exon 20 which occurs in almost 50–60% of patients that develop disease progression after administration of EGFR‐TKIs.^8^

Osimertinib (AZD9291), a third generation EGFR TKI, is a mono‐anilino‐pyrimidine small molecule. It has also been approved for second‐line treatment of advanced NSCLC positive for EGFR‐T790M mutations.^9^, ^10^ Osimertinib was developed to target double‐mutant (activating mutation + T790M) forms of EGFR resistant to first‐ and second‐generation EGFR TKIs.^10^ Prospective randomized controlled trials confirmed that osimertinib could improve the treatment response in patients carrying the EGFR T790M mutation.^11^ The EGFR T790M point mutation (T790M) is the most common drug resistant mechanism in EGFR mutation‐positive NSCLC patients. Osimertinib is a third‐generation EGFR‐TKI approved by the US FDA for the treatment of EGFR‐T790M‐positive NSCLC with a reported response rate greater than 60%^5^. Although EGFR‐TKIs can improve the prognosis of NSCLC with effective mutation, it also has drug‐related toxicities which include severe reactions such as rashes, oral mucositis, hepatotoxicity and interstitial lung disease (ILD). The incidence of drug‐related ILD in different EGFR‐TKIs ranged from 0 to 5.3%. The incidence rate of ILD caused by osimertinib remains unclear because of the small sample size reported in the literature. In the case reported here, the patient developed severe ILD after taking osimertinib and finally died of ILD and associated complications. This case indicates that a serious adverse complication such as interstitial lung disease, caused by Osimertinib, should be given urgent attention and the correct treatment administered as early as possible.

## Disclosure

The authors do not report any conflict of interest.
